# Formation of Self-Healing Granular Eutectogels through Jammed Carbopol Microgels in Supercooled Deep Eutectic Solvent

**DOI:** 10.1021/acs.langmuir.4c02069

**Published:** 2024-07-30

**Authors:** Karthi Keyan Arjunan, Chun-Yun Weng, Yu-Jane Sheng, Heng-Kwong Tsao

**Affiliations:** † Department of Chemical and Materials Engineering, National Central University, Taoyuan 32001, Taiwan; ‡ Department of Chemical Engineering, National Taiwan University, Taipei 10617, Taiwan

## Abstract

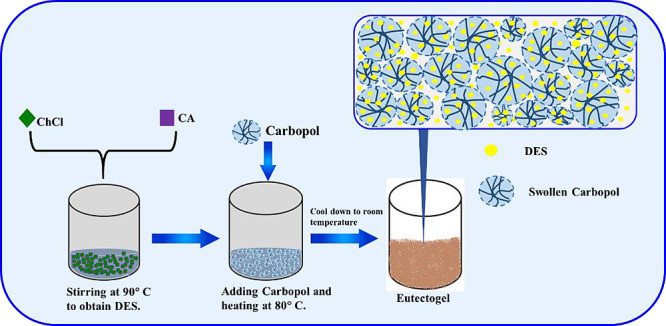

Typically, gel-like materials consist of a polymer network structure in a solvent. In this work, a gel-like material is developed in a deep eutectic solvent (DES) without the presence of a polymer network, achieved simply by adding microgels. The DES is composed of choline chloride and citric acid and remains stably in a supercooled state at room temperature, exhibiting Newtonian fluid behavior with high viscosity. When the microgel (Carbopol) concentration exceeds 2 wt %, the DES undergoes a transition from a liquid to a soft gel state, characterized as a granular eutectogel. The soft gel characteristics of eutectogels exhibit a yield stress, and their storage moduli exceed the loss moduli. The yield stress and storage moduli are observed to increase with increasing microgel concentration. In contrast, the ion conductivity decreases with increasing microgel concentration but eventually levels off. Because the eutectogel can dissolve completely in excess water, it is a physical gel-like material, attributed to the densely packed structure of microgels in the supercooled DES. Due to the absence of networks, the granular eutectogel has the capability to self-heal simply by being pushed together after being cut into two pieces.

## Introduction

Deep eutectic solvents (DESs) are eutectic mixtures commonly prepared using two or more substances, which consist of hydrogen bond donors (HBD) and hydrogen bond acceptors (HBA).^1−3^ Most of the DESs contain one ionic species and are dominated by strong hydrogen bonding interactions, rendering them hydrophilic.^4−6^ For example, Type III DES is a mixture of quaternary ammonium salt and a hydrogen bond donor.^7,8^ DESs are nontoxic or have low toxicity, and they are cost-effective, easy to prepare, and biodegradable in nature.^9,10^ Therefore, DESs can serve as good alternative solvents by replacing toxic and volatile organic solvents to reduce the environmental burden. Additionally, DESs are noteworthy green solvents due to their unique physical properties, including conductivity and surface tension.^11,12^ Due to these properties, DESs are applicable in various fields such as catalysts, electrochemistry, and many industrial applications.^13,14^ Specific examples include green gold separation,^15^ green self-adhesive tapes,^16^ and green and efficient cryopreservation.^17^

Similar to a hydrogel with a polymer network in water, a eutectogel, exhibiting soft gel behavior, can be developed using a network in DES, which has attracted considerable attention.^18−20^ An example of a chemical eutectogel is the cross-linked poly(acrylic acid) (PAA) network in a DES containing choline chloride (HBA) and urea (HBD), formed after the polymerization of acrylic acid monomers.^21,22^ In contrast, a physical eutectogel example is the poly(vinyl alcohol) (PVA) network in a DES containing metal salts (HBA) such as lithium chloride or zinc chloride, and ethylene glycol or glycerol (HBD).^23,24^ PVA was dissolved in DES to form eutectogel through the establishment of multi hydrogen bonds and coordination bonds with metal ions. The noncovalent cross-linking points in this physical eutectogel are associated with the small crystalline domains of PVA.^25,26^ Unlike hydrogels, eutectogels exhibit intrinsic characteristics such as low vapor pressure and high thermostability^27−29^ associated with the properties of DES. Due to their outstanding properties, including flexibility, ionic conductivity, and stimulus responsiveness, eutectogels are utilized in various applications such as strain sensing,^30,31^ electrochemistry,^32,33^ wastewater treatment,^34^ and electrochromic materials.^35^

Microgels are internally cross-linked macromolecules that have been used as rheological modifiers.^36^ Synthetic microgels, such as Carbopol, share similarities with dendrimers or hyperbranched polymers, characterized by their high molecular weight.^37,38^ An aqueous dispersion of microgels can exhibit soft gel behavior, similar to typical hydrogels, despite the absence of a network. These soft materials utilize microgels (micron-sized hydrogels) as fundamental building blocks to exhibit gel-like (soft gel) behavior, hence referred to as “granular hydrogel”.^39^ As microgels are densely packed and move in conjunction with adjacent microgels, they exist in a jammed state.^40,41^ In the case of jammed microgels, physical interactions immobilize granular hydrogels, leading to the manifestation of soft gel behavior in the entire system. When a sufficiently high stress is applied to the sample, it changes to a liquid state and starts to flow due to the movement of microgels.^42,43^ Below the yield stress, the microgel volume may deform elastically, and as a result, there is no movement of individual microgels.^44,45^ Certainly, the sample recovers its soft gel behavior when the applied stress is removed.^46,47^

Both chemical and physical eutectogels have been reported in the literature, and their gel-like behavior originates from the polymer network. In the case of physical eutectogels, it is essential to identify suitable polymers that can dissolve in the chosen DES. Moreover, the polymer cannot fully dissolve in the DES, as partial crystallization must occur to establish the necessary cross-linking points.^25^ Consequently, only a few physical eutectogel systems have been reported thus far.^48−50^ In this work, we propose another strategy for developing physical eutectogels without resorting to any network. Physical eutectogels are fabricated by utilizing low-cost commercial microgels (Carbopol) in conjunction with a DES containing choline chloride (HBA) and citric acid (HBD). The eutectic point of this DES was approximately 76 °C at a 3:1 molar ratio. However, a recent report indicates that it remains in a liquid state stably at room temperature below the eutectic point.^51^ Therefore, the combination of choline chloride and citric acid is utilized as the solvent for gel-like materials. The influence of Carbopol concentration on the rheological behavior of the eutectogel is systematically explored. After delineating the changes in rheological properties, the transition from liquid to soft gel behavior is characterized. Additionally, the yield stress and electric conductivity are both studied. Finally, the mechanism of physical eutectogel formation is investigated, and its relation to the jammed structure is established.

## Experimental Methods

### Materials

Choline chloride (ChCl) 98% and citric acid monohydrate (CA) 99% were purchased from Alfa Aesar Co., while Carbopol 2020 was obtained from Top Rhyme International Co., Taiwan. The melting points of ChCl and CA are 302 and 156 °C, respectively.

### Preparation of DES and Eutectogel

**Scheme 1:**
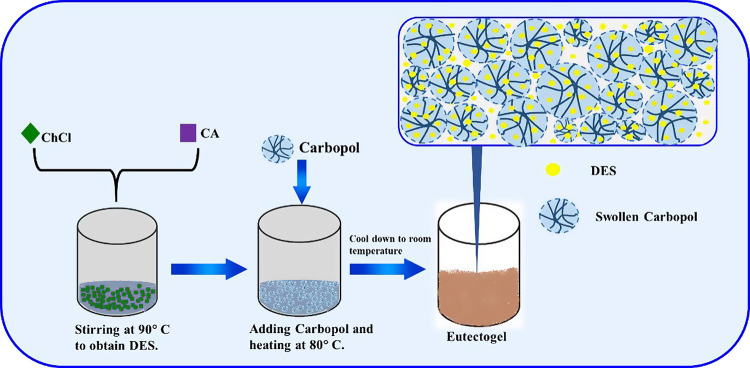
Synthesis Process of the Physical Eutectogel

Type III DES was prepared using ChCl as HBA and CA as HBD, with the molar ratio of ChCl to CA fixed at 3:1. The mixture was heated at 90 °C for 30 min in an oil bath, after which a small amount of Carbopol (0.5–3.5 wt %) was added to the solution and thoroughly mixed. The three-component mixture was then heated at 80 °C for another 30 min. The solution was then cooled to room temperature to obtain either liquid or soft gel materials. The eutectogel was obtained at sufficiently high concentrations of Carbopol, as illustrated in Scheme 1.

### Rheological Analysis

Rheological analysis of the prepared eutectogel was conducted using a modular compact rheometer (Anton Paar MCR 92, USA) with a parallel (regular smooth and rough) plate of 25 mm diameter. The storage modulus (*G*′) and loss modulus (*G″*) were determined through an oscillatory stress sweep ranging from 0.01 to 1000% at a frequency of 1 Hz. Apparent viscosity was measured using a continuously ramped shear rate from 0 to 50 s^–^^1^. All measurements were conducted at room temperature.

### Conductivity Measurement

The resistances (ionic conductivities) of the eutectogels were determined using electrical impedance spectroscopy. The sample resistance (*R*) was obtained from the intercept between the impedance curve and the real axis. The ionic conductivity (σ) is calculated using the formula σ = *d*/(*R* × *l* × *w*), where *d* represents the distance between two metals, *l* is the length of the metal immersed in the sample, and *w* is the width of the metal.

## Results and Discussion

### Transition from Liquid to Soft Gel Behavior

**Figure 1:**
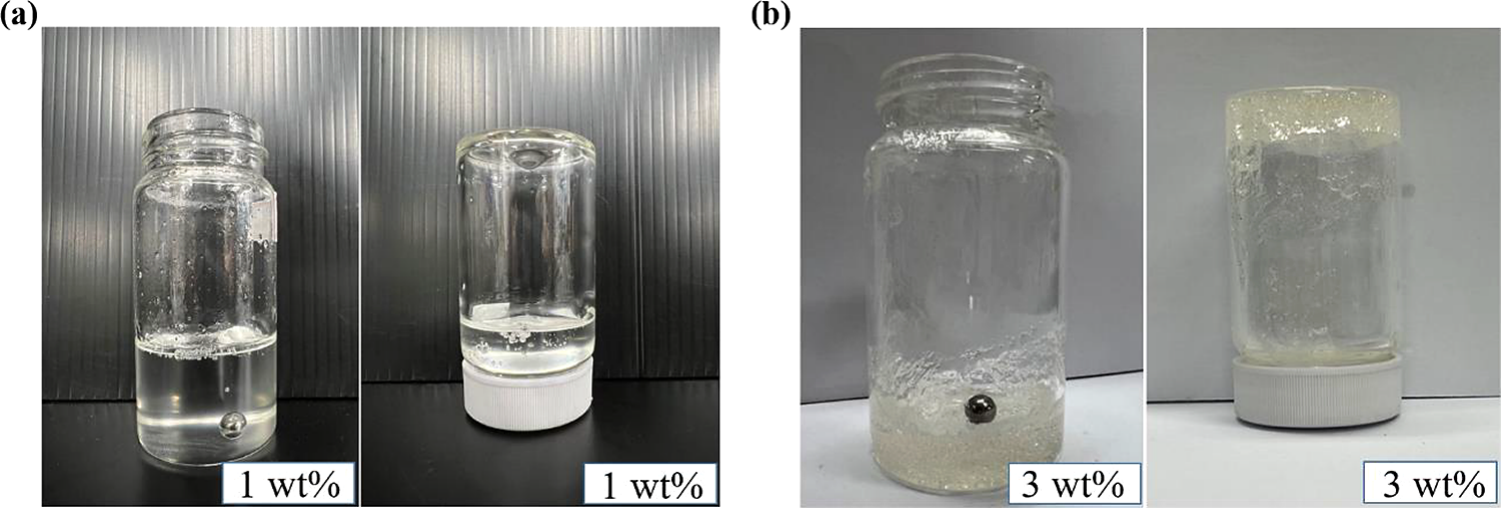
Falling ball and inverted tube tests for ChCl:CA = 3:1 DES with (a) 1 wt % and (b) 3 wt % of Carbopol.

The 3:1 mixture of ChCl and CA yields a supercooled DES liquid at room temperature, displaying Newtonian fluid behavior with high viscosity. This DES solvent is capable of dispersing Carbopol, a microgel. After the addition of Carbopol, the DES containing Carbopol exhibits viscoelastic behavior. However, the mechanical response of this mixture depends on the amount of Carbopol added. The qualitative characteristics of the viscoelastic mixture can be easily examined through falling ball and inverting tube tests. The former test involves a steel ball with a diameter of 0.6 cm and a load-bearing capacity of 0.49 g. The results, illustrated in Figure 1, are presented for two Carbopol concentrations: 1 and 3% by weight. As shown in Figure 1a for the lower Carbopol concentration, the steel ball falls to the bottom while the sample flows downward along the inverted tube. These outcomes indicate that the DES with 1 wt % Carbopol behaves as a viscoelastic liquid, rather than a viscoelastic solid. On the contrary, at higher Carbopol concentrations, the steel ball rests at the top while the liquid mixture remains static at the bottom of the inverted tube (with no downward flow), as shown in Figure 1b. These results suggest that the DES with 3 wt % Carbopol exhibits soft gel behavior. Note that the stability of this gel-like material is reasonably good. When exposed to a highly humid environment (RH ∼ 84% at 25 °C) for 12 h, the weight of the sample (3 wt % Carbopol) increases by only 10 wt % due to water absorption.

**Figure 2:**
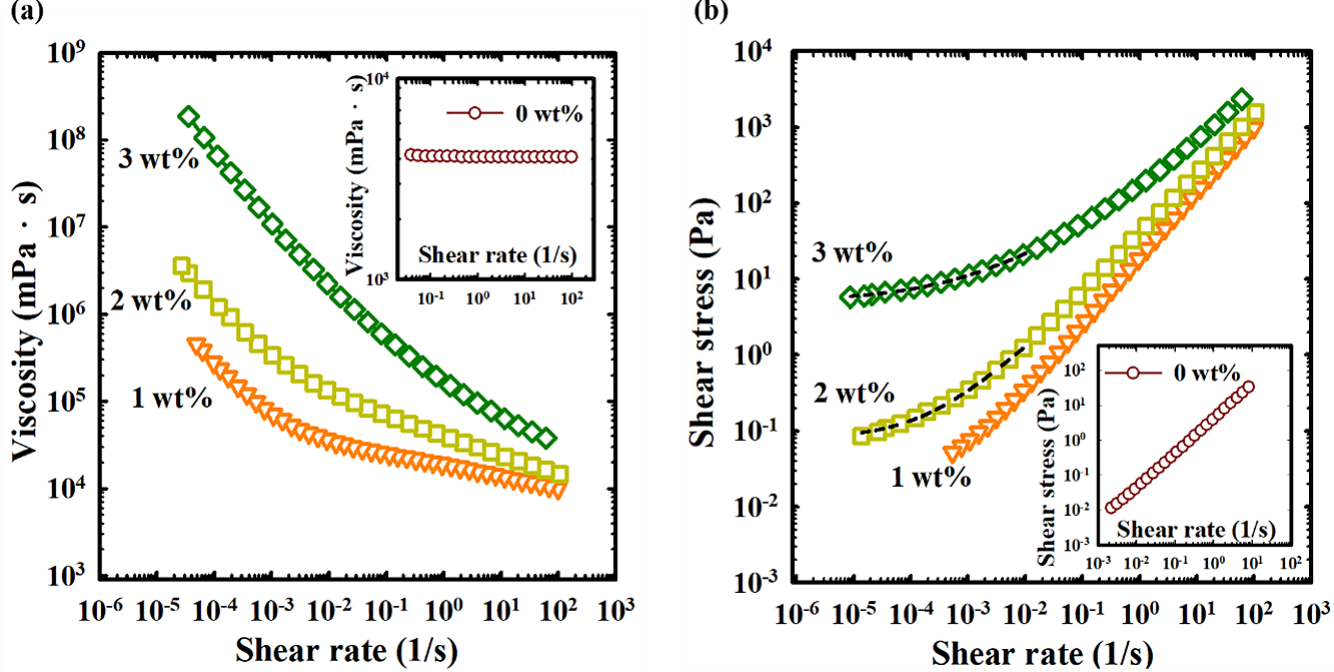
(a) The flow curve of Carbopol-containing DESs (1–3 wt %). (b) The yield stress determined from the Herschel–Bulkley equation (represented by the dash curve) for 2 and 3 wt % of Carbopol. Both insets demonstrate the behavior of pure DES.

To quantify the viscoelastic behavior of Carbopol-containing DESs, rheological measurements were performed to obtain the flow curves. Figure 2 illustrates the rheological properties of the Carbopol-containing DESs prepared by dispersing different amounts of Carbopol, showing the variation of viscosity with shear rate in Figure 2a. In the absence of Carbopol (0 wt %), the viscosity remains constant (as shown in the inset), indicating that the DES behaves as a Newtonian fluid. However, after dispersing Carbopol, the viscosity grows dramatically and displays shear-thinning behavior. The apparent viscosity at a specific shear rate increases with the Carbopol content. The rheological results, presented in the form of a plot showing shear stress against shear rate (depicted in Figure 2b), reveal distinct characteristics of Carbopol-containing DESs. When the Carbopol concentration exceeds 2 wt %, a finite value of shear stress is observed as the shear rate approaches zero, indicating the presence of yield stress. In contrast, for 0 wt % Carbopol shown in the inset, the shear stress is linearly proportional to the shear rate, a typical signature of Newtonian fluid. The yield stress represents the minimum shear stress or external force required to disrupt the structure of the material at rest and initiate its flow.^28^ According to the Herschel–Bulkley equation, the yield stress is estimated as τ_0_ = 7.15 × 10^–2^ Pa for 2 wt % and τ_0_ = 5.20 Pa for 3 wt %. The presence of the yield stress reveals that soft gel behavior emerges when a sufficiently high amount of Carbopol is present.

The addition of Carbopol, functioning just as crystallization nuclei, in supercooled DES may result in the liquid–solid transition. However, it does not apply to our DES because the eutectogel sample remains unchanged even after at least four months. It is still soft and deformable, unlike a rigid solid, the liquid behavior illustrated in Figure 2 for viscosity is consistently observed. We have utilized DES (choline chloride and ethylene glycol), a liquid at room temperature, to produce the granular eutectogel by incorporating Carbopol. However, the mechanical strength of the resulting gel is relatively weak.

**Figure 3:**
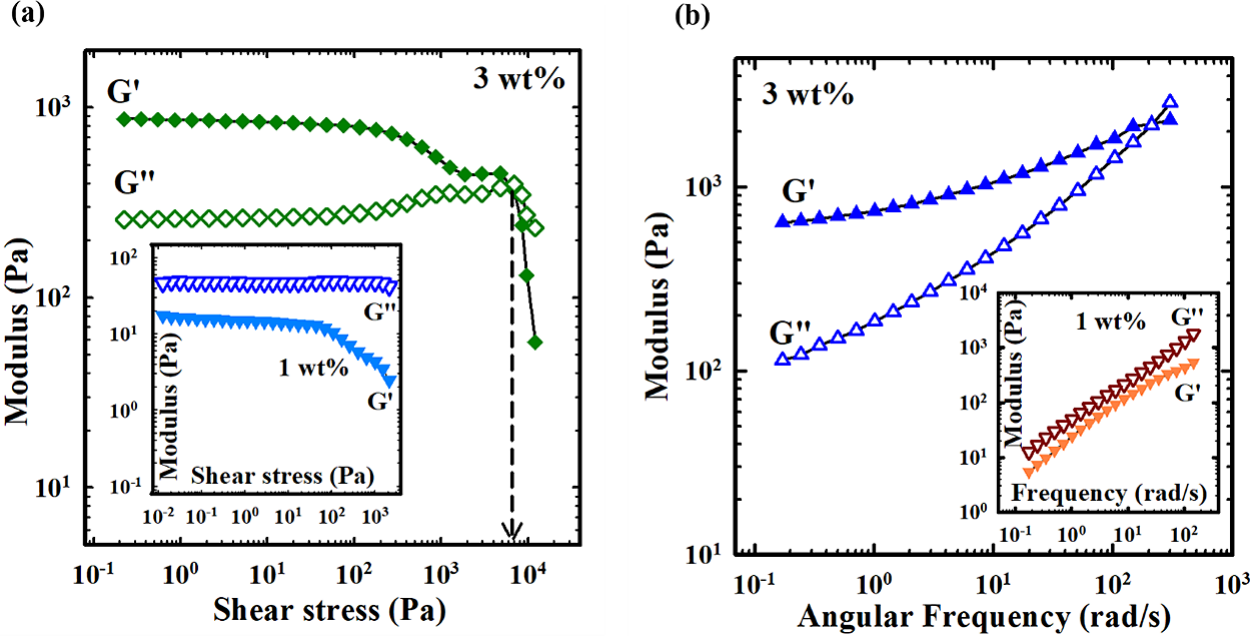
Mechanical moduli of Carbopol-containing DESs (1 and 3 wt %) for (a) amplitude and (b) frequency sweep tests. In the inset, the Carbopol concentration is 1 wt %.

The viscoelastic behavior of Carbopol-containing DESs can be further analyzed through oscillation sweeps of amplitude and frequency. The variation of the mechanical moduli (storage modulus *G*′ and loss modulus *G″*) with amplitude or frequency is then obtained to understand the rheological behavior, as illustrated in Figure 3. The amplitude sweep is depicted in Figure 3a for a fixed frequency 1 rad/s. As illustrated in the inset of Figure 3a, one has *G″* > *G*′, indicating a liquid behavior for 1 wt % Carbopol. On the contrary, the storage modulus becomes greater than the loss modulus (*G*′ > *G″*) for 3 wt % of Carbopol as shown in Figure 3a, revealing a soft gel behavior. Nonetheless, for sufficiently large amplitudes (strain or stress), one still has a liquid behavior (*G″* > *G*′) and the crossover point indicates the dynamic yield stress. The frequency sweep is depicted in Figure 3b for a fixed strain 1%, and the results are similar to those associated with the amplitude sweep. The liquid behavior (*G″* > *G*′) is observed for 1 wt % Carbopol (see inset), whereas soft gel behavior (*G*′ > *G″*) is seen for 3 wt %. The gel-like behavior is generally recognized as long as *G*′ > *G″* at low frequencies. Nevertheless, the liquid behavior still appears at high frequencies for 3 wt % Carbopol, revealing the feature of weak gel. To examine the effect of hydrogel “slip”,^52^ we also conducted experiments using both regular (smooth) and rough steel plates. The results were very similar, at least qualitatively. To investigate the thermal stability of the prepared eutectogel, various temperatures including 40, 50, and 60 °C were examined. The results were found to be essentially the same as those observed at 25 °C.

### Effect of Carbopol Concentration

**Figure 4:**
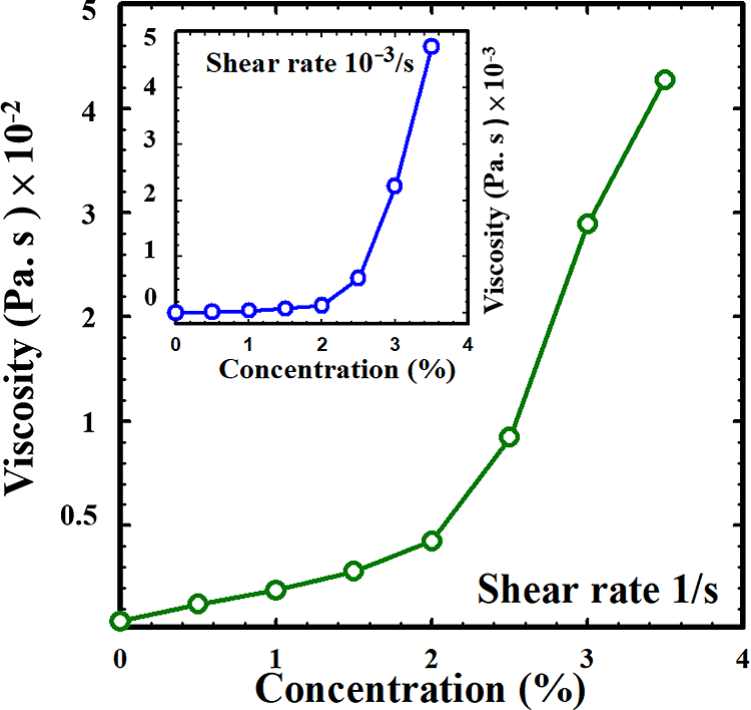
Variation of the apparent viscosity with the Carbopol concentration at the shear rate 1/s. The result at a lower shear rate 10^–3^/s is shown in the inset.

The results mentioned above reveal that the behavior of the DES mixture varies significantly depending on the amount of Carbopol added. To demonstrate the influence of Carbopol concentration on the flow behavior, the apparent viscosity associated with a specific shear rate (1/s and 10^–3^/s) is plotted against the concentration, as shown in Figure 4. It is evident that the apparent viscosity increases with the Carbopol concentration, indicating that the addition of Carbopol enhances the resistance of DESs to deformation. At lower Carbopol concentrations, the apparent viscosity at the shear rate 1/s gradually increases. However, once the Carbopol concentration exceeds 2 wt %, it begins to rise rapidly. The similar phenomenon has also been observed for a low shear rate of 10^–3^/s, as illustrated in the inset of Figure 4. The rapid increase in viscosity after reaching a critical Carbopol concentration implies that the Carbopol-containing DES transitions from a liquid to a soft gel state. The critical Carbopol concentration is approximately 2 wt %, beyond which the mixture exhibits a gel-like behavior.

**Figure 5:**
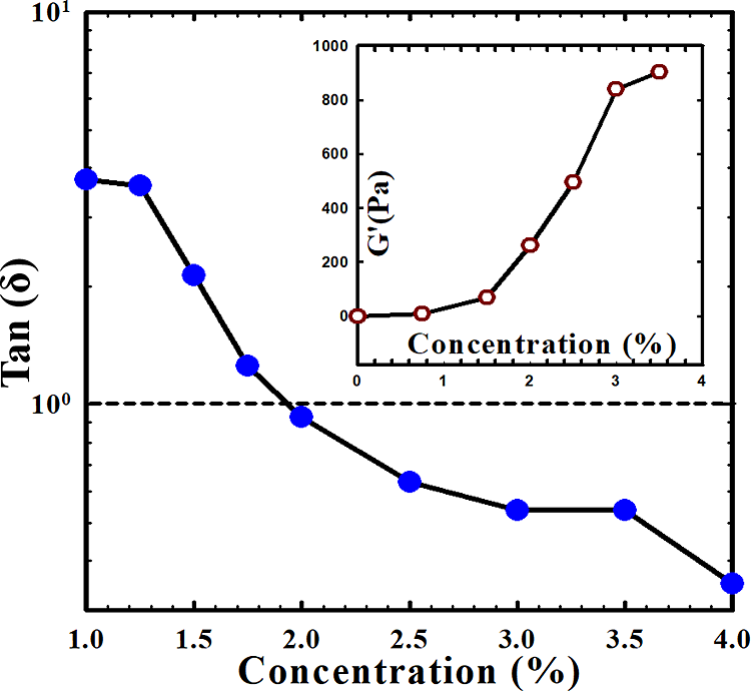
Variation of tan(δ) with Carbopol concentration is depicted. In the inset, the variation of *G*′ at a frequency of 1 rad/s and amplitude of 1% with Carbopol concentration is shown.

**Figure 6:**
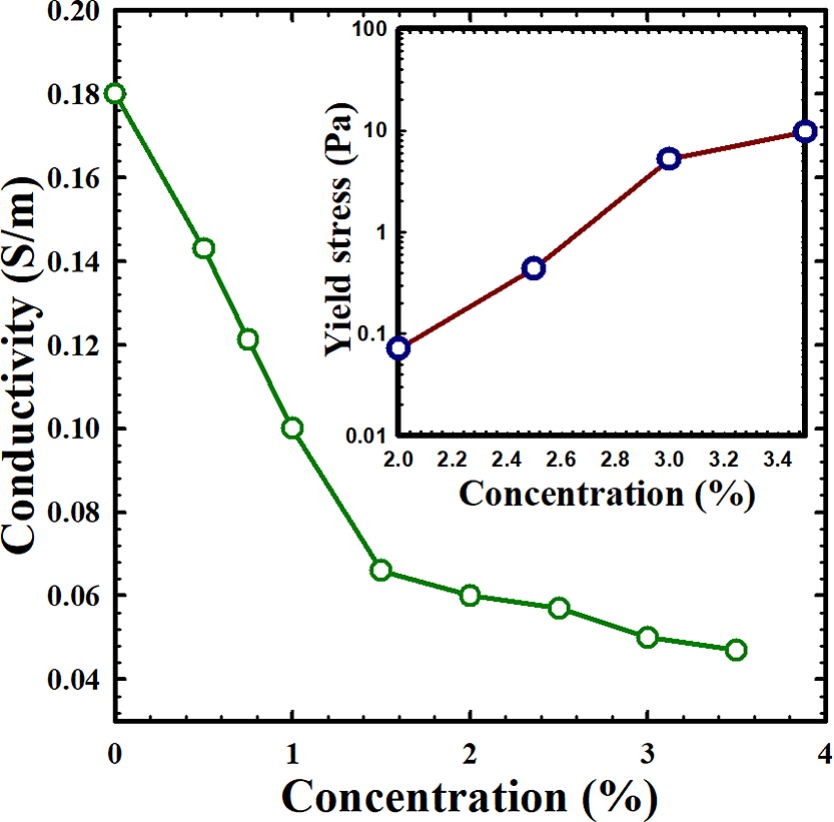
Variation of the conductivity with the Carbopol concentration. The variation of the yield stress (eutectogel) with the Carbopol concentration is shown in the inset.

The influence of the Carbopol concentration can also be demonstrated by the elastic properties of the mixture. As depicted in the inset of Figure 5, the storage modulus (at a strain of 1% and a frequency of 1 rad/s) increases with the Carbopol concentration. It starts to rise rapidly after reaching approximately 2 wt %, indicating that the Carbopol-containing DES tends to transition to a soft gel state. Quantitatively, the transition from liquid to soft gel behavior can be characterized by tan(δ) = *G″*/*G*′, which reveals the contribution of the elastic (recoverable) behavior. Figure 5 shows that tan(δ) decreases with an increase in Carbopol concentration, indicating that the recoverable behavior becomes more significant as more Carbopol is incorporated. It is observed that tan(δ) falls below unity (*G″* < *G*′) when the Carbopol concentration exceeds 2 wt %. This result confirms that, when the Carbopol concentration surpasses the critical value of 2 wt %, the DES transitions to a soft gel state due to the dominance of the elastic component over the viscous component. Consequently, the yield stress, indicative of a soft gel property, emerges. The variation of yield stress with Carbopol concentration is depicted in the inset of Figure 6. Evidently, the yield stress increases gradually with the addition of more Carbopol microgels.

In addition to commendable mechanical properties, ionic conductivity is a crucial parameter for eutectogels. It serves as a fundamental measure, quantifying the materials’ ability to conduct ions and reflecting the tendency of ion movement within solutions and solid substances. Figure 6 illustrates the variation in ion conductivity of the eutectogel with different amounts of added Carbopol (ranging from 0.5 to 3.5 wt %). In the absence of Carbopol (0 wt %), the DES exhibits an ionic conductivity of 0.18 S/m. With the addition of a small amount of Carbopol to the DES, the ionic conductivity starts to decline, even though the mixture retains a liquid behavior. This result can be attributed to the increase in viscosity, as depicted in Figure 4, which in turn elevates the resistance to ion movement. With an increase in Carbopol concentration, the apparent viscosity of the liquid mixture grows, further hindering ion motion and resulting in lower conductivity. As the soft eutectogel forms, the hindrance to the transport of ions in dense and jammed structures approaches its maximum. The ionic conductivity decreases from 0.18 to 0.06 S/m. Further addition of Carbopol strengthens the mechanical properties of the eutectogel but introduces slight resistance to ion movement. Therefore, the ion conductivity decreases only from 0.06 to 0.047 S/m with the increase in Carbopol concentration from 2.0 to 3.5 wt %. Despite the decrease in ionic conductivity with increased Carbopol amount, the conductivity remains high, reaching 0.05 S/m at 3 wt %. This level of conductivity is still suitable for fabricating wearable sensors.^42,48,53,54^

### Mechanism of Physical Eutectogel: Self-Healing and Jammed Structure

**Figure 7:**
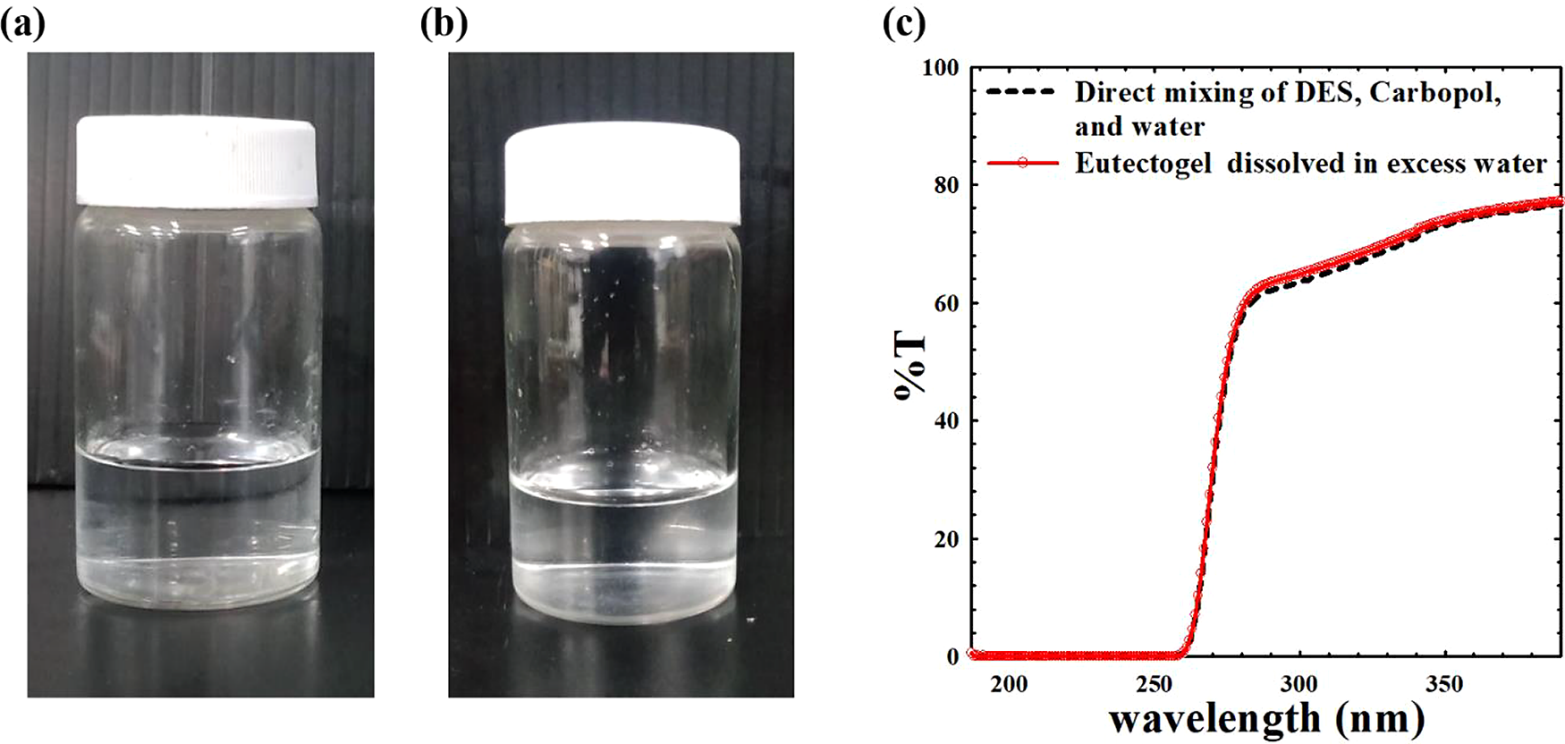
(a) A clear solution is formed as the eutectogel is dissolved in excess water, indicating its complete dissolution. (b) A mixture replicating the composition in (a) is prepared by direct mixing at room temperature. (c) The quantitative comparison between the two samples in (a) and (b) through UV–vis analysis.

The eutectogel, displaying soft gel behavior, becomes evident when the Carbopol concentration exceeds 2 wt %. The preparation process involves direct mixing of the components at 80 °C, with no apparent chemical reaction involved in the formation of the gel network. To investigate whether a reaction occurs during gel formation, the eutectogel is immersed in an abundance of water (95 wt %). If a permanent (chemical or physical) network structure is formed, it cannot be dissolved in water at room temperature, even if the solvent itself is soluble in water. This is because the large, strong cross-linking network cannot be dissolved at all. In fact, a loose, cotton-like structure associated with the covalent cross-linking network will appear when the chemical eutectogel is immersed in water, given that both DES and Carbopol are soluble in water. However, a clear solution, as shown in Figure 7a, is observed when the eutectogel is immersed in water, indicating its complete dissolution. To confirm these dissolution results, a mixture containing DES, Carbopol, and water, replicating the composition in Figure 7a, is prepared by direct mixing at room temperature for comparison. The final outcome is also a clear solution, as depicted in Figure 7b. Note that the Carbopol microgel is transparent under the optical microscope, indicating that most of the swollen microgel contains water. As a result, both Mie and Rayleigh scattering are not significant.^55−57^ Despite their similar appearance, a quantitative comparison is necessary to assess their similarity. Figure 7c illustrates the quantitative comparison between the two aforementioned samples through UV–vis analysis. Evidently, their spectra overlap, indicating that the final aqueous solutions are independent of the preparation methods (path). Therefore, our eutectogel, formed through Carbopol addition, is a physical gel rather than a chemical gel. The Carbopol microgel can be regarded as a soft sphere. When its concentration surpasses a certain threshold, these soft spheres become crowded, resulting in the formation of a jammed structure and exhibiting soft gel behavior. Typically, eutectogels exhibit network structures, and their morphology can be examined using SEM images for the dry sample after replacing DES with water and subsequent drying. However, in our study, the physical eutectogel dissolved completely in water, making it impossible to ascertain its morphology.^58,59^

**Figure 8:**
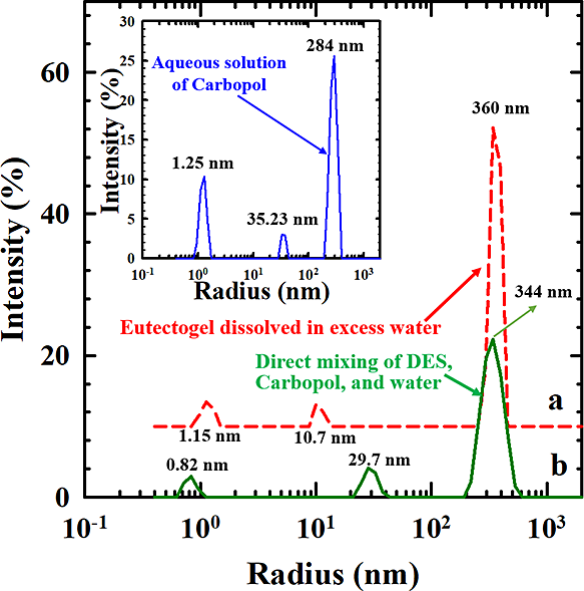
Particle size distribution of Carbopol microgels for the samples depicted in Figure 7a,b is determined by dynamic light scattering. The inset illustrates the size distribution for the aqueous solution of Carbopol.

After the dissolution of physical eutectogels in water, Carbopol microgels are expected to behave as isolated nanoparticles and disperse in water due to the absence of chemical reactions between them. That is, the particle size distribution of our physical eutectogel in water is expected to be similar to that of Carbopol dissolved in water or the mixture formed by directly mixing DES, Carbopol, and water. As depicted in the inset of Figure 8, the particle size distribution obtained from dynamic light scattering for the aqueous solution of Carbopol reveals three characteristic peaks. Similar results are observed for both the physical eutectogel dissolved in water and the mixture formed by direct mixing, as demonstrated in Figure 8. Evidently, the mean size of most microgels is approximately 300 nm. However, a small fraction of microgels exhibits a mean size of about 30 nm. The peak around 1 nm is likely attributed to impurities. Their *z*-average diameter and polydispersity are similar, estimated to be approximately 360 nm and 0.82, respectively. The similarity among these three particle size distributions reiterates that the eutectogels developed through the addition of Carbopol are not the result of chemical reactions involving Carbopol. The gel-like behavior (*G*′ > *G″*) of the eutectogel, as observed in Figures 1 and 3, is achieved simply by dispersing a sufficient number of microgels in DES. According to Figure 8, no chemical reaction occurs among the Carbopol particles. Therefore, the granular eutectogel’s behavior is not due to a chemical or physical network but is instead caused by the jammed structure of swollen microgels (physical interaction).

**Figure 9:**
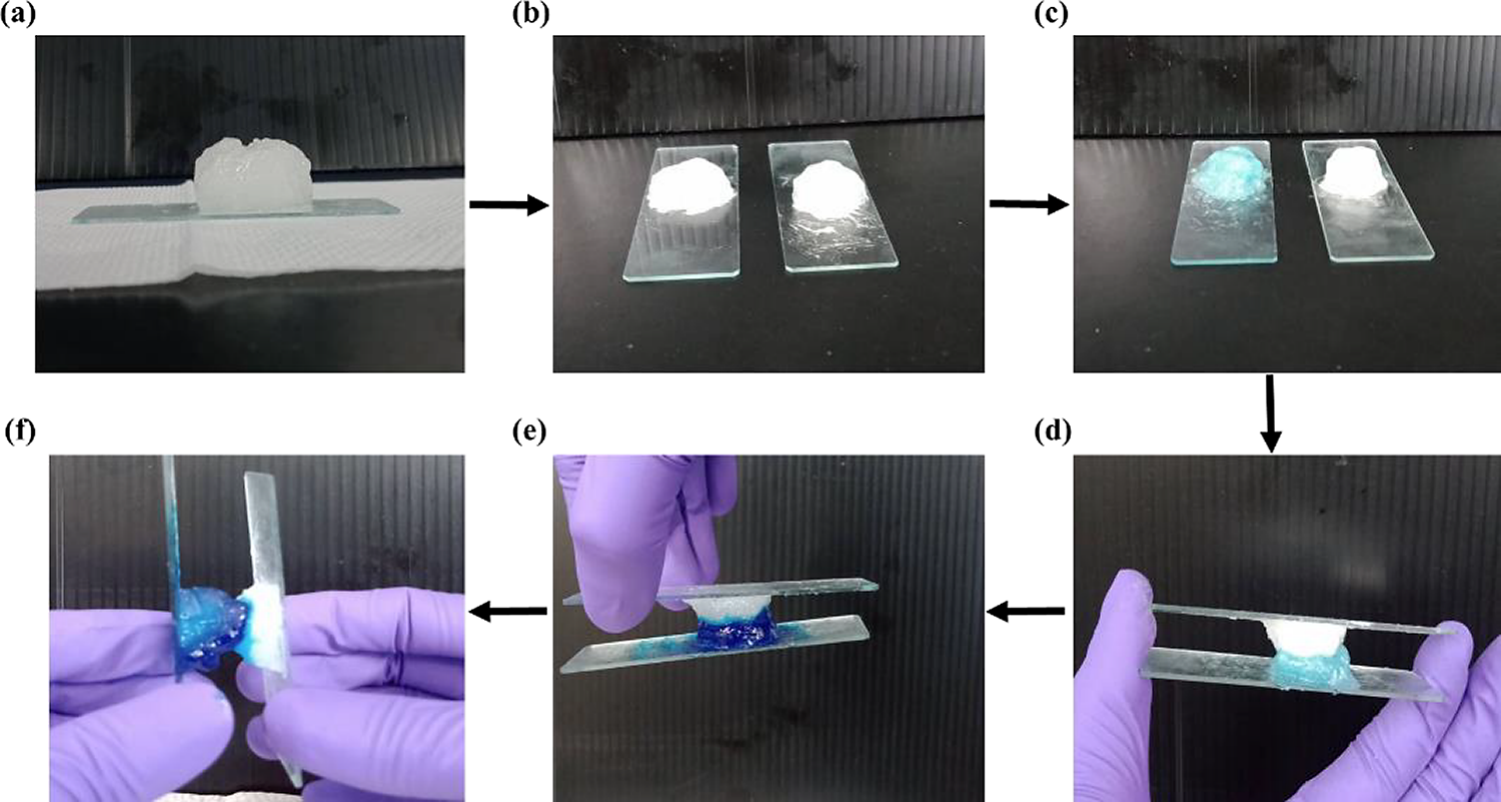
Illustration of the self-healing process of the prepared eutectogel: (a) initial state, (b) separation with each part deposited on a glass slide, (c) dyeing the left part with methylene blue, (d) recombination for 6 h, (e) showcasing how self-healing prevents the falling of the lower part, and (f) demonstrating the resistance to separation by weak pull.

The physical origin of our DES-based gel-like material, as opposed to a chemical one, is further evident through its unique self-healing property. The sample can be cut into two parts and then recombined simply by bringing them into contact again, as demonstrated in Figure 9, without the need for any additional means. The self-healed eutectogel, resulting from the contact of two fractured Carbopol-containing eutectogels for 6 h, exhibited the capability to endure substantial tensile deformation and remarkable durability without cracking. It is worth emphasizing that no heating treatment or addition of other components, such as Carbopol, is required to facilitate self-healing. The self-healing process can be accomplished solely at room temperature within 6 h with the assistance of a push from gravitational force. The self-healing result suggests that the separation process of the eutectogel does not involve breaking the chemical network bonds, which cannot be restored through physical processes such as pushing the parts together. That is, our eutectogel is formed without the creation of a chemical network, making it a physical gel.

The liquid DES transforms into a soft gel material upon the addition of a sufficient amount of microgels. The formation of hydrogen bonds between different microgels is possible, but they are too weak to serve as cross-linking points typically associated with multiple hydrogen bonds. This absence of a physical network is evident, as the material can completely dissolve or disperse in water. Clearly, our eutectogel is not formed through either a chemical or physical network. Without a network to sustain externally applied forces, the soft gel material must rely on its jammed structure. A jammed structure with dense packing refers to a highly concentrated and immobilized arrangement of particles within a system. Even at a low concentration of 3 wt %, the Carbopol microgel can swell significantly in the solvent, expanding from a few times up to hundreds or even a thousand times its dry volume.^60,61^ This means that a swollen microgel contains a very small weight fraction of polymer but a large weight fraction of solvent. As a result, a jammed structure can form. In other words, the swollen microgels are densely packed, making them resistant to easy deformation, and they tend to restore their shape after being deformed. Due to the jammed structure, the microgel-containing DES displays gel-like behavior (*G*′ > *G″*) and can be termed a “granular eutectogel”. It is worth mentioning that, at room temperature, the ChCl-CA based DES is expected to be in a solid state, yet it behaves like a Newtonian liquid. Essentially, the DES is in a supercooled state with very high viscosity.^46^ Consequently, the mechanical strength of our physical eutectogel is likely significantly enhanced by the synergistic interactions between densely packed microgels and the supercooled DES.

## Conclusions

The gel-like materials commonly used in pharmaceuticals and the food industry typically consist of a polymer network structure in a solvent. However, in this work, we have developed a soft gel material based on the DES without the presence of a polymer network. The prepared DES is composed of ChCl and CA with a molar ratio of 3:1, and it displays Newtonian fluid behavior with high viscosity. This DES is in a stably supercooled state at room temperature, even though the system temperature is significantly below the deep eutectic point. When a small amount of microgels (Carbopol) is added, the mixture exhibits non-Newtonian fluid behavior with shear-thinning characteristics. As the Carbopol concentration exceeds 2 wt %, the mixture transitions from a liquid to a soft gel state at room temperature. The soft gel Carbopol-containing DES can be classified as a granular eutectogel, representing a green, low-cost, and low-toxicity material.

The soft gel characteristics of granular eutectogels are qualitatively demonstrated through falling ball and inverted tube tests. Quantitatively, they exhibit a yield stress, and their storage moduli exceed the loss moduli. The yield stress and storage moduli are observed to increase with an increase in microgel concentration. In contrast, the ion conductivity is observed to decrease with an increase in microgel concentration but eventually levels off, indicating that the hindrance to ion movement is minimally affected by the dense packing of microgels. The eutectogel is considered a physical gel-like material as it can dissolve in excess water without leaving any residues of networks. Additionally, the size distribution of microgels remains essentially unchanged before and after the formation of eutectogels. Consequently, the creation of our granular eutectogels is attributed to the jammed structure of microgels in supercooled DES. Due to the absence of networks, the granular eutectogel has the capability to self-heal simply by being pushed together after being cut into two pieces. This Carbopol-containing eutectogel holds promising potential for applications in gel electrolytes, wearable electronic devices, and health monitoring sensors.
